# Psychosocial Determinants of Childbirth Fear Among Asian Women: A Scoping Review

**DOI:** 10.3390/healthcare13131535

**Published:** 2025-06-27

**Authors:** Aida Kalok, Ixora Kamisan Atan, Shalisah Sharip, Nazarudin Safian, Shamsul Azhar Shah

**Affiliations:** 1 Department of Public Health Medicine, Faculty of Medicine, Hospital Canselor Tuanku Muhriz, Universiti Kebangsaan Malaysia, Jalan Yaacob Latif, Kuala Lumpur 56000, Malaysia; aidahani.mohdkalok@hctm.ukm.edu.my (A.K.);; 2 Department of Obstetrics & Gynaecology, Faculty of Medicine, Hospital Canselor Tuanku Muhriz, Universiti Kebangsaan Malaysia, Jalan Yaacob Latif, Kuala Lumpur 56000, Malaysia; 3 Department of Psychiatry, Faculty of Medicine, Hospital Canselor Tuanku Muhriz, Universiti Kebangsaan Malaysia, Jalan Yaacob Latif, Kuala Lumpur 56000, Malaysia

**Keywords:** Asia, childbirth, fear, pregnancy, psychology, social support

## Abstract

**Background:** Fear of childbirth (FOC) or tokophobia has a detrimental impact on women during and after pregnancy. Childbirth fear is multidimensional and may differ across nations and cultures. We aimed to determine the psychosocial determinants of tokophobia among Asians. **Methods:** We conducted a systematic literature search using the PubMed, Scopus, and Web of Science databases in September 2023. Included were original English-language articles that focused on Asian cohorts. We identified independent variables associated with maternal childbirth fear based on multivariable logistic and linear regression analysis. **Results:** Twenty-three studies are discussed in this review. We categorized the factors into (1) psychiatry, (2) psychology, (3) perception and experience, (4) relationships and support, (5) spirituality, and (6) COVID-19. The studies involved 10,538 women with overall FOC prevalence ranging between 56.6% to 82.1%. Maternal history of psychiatric disorder, depression, and anxiety were positive predictors of childbirth fear. Childbirth self-efficacy protects Asian mothers against tokophobia. A reduced level of fear was associated with higher maternal psychological and spiritual well-being, as well as stronger maternal resilience. Spousal and social support alongside good family function were shown to exert a protective effect against childbirth fear. Intimate partner abuse was associated with an increased risk of tokophobia in women. Studies during the pandemic indicated that maternal fear, obsession, and anxiety about COVID-19 were positively correlated to fear of childbirth. **Conclusions:** Childbirth fear among Asian women is greatly influenced by various psycho-social factors. More culturally driven research is needed to help develop relevant interventions that will enhance maternal psychological and spiritual well-being and reduce the fear of childbirth.

## 1. Introduction

The experience of childbirth is a complex individual life process with significant subjective psychological and physiological elements that are impacted by social and environmental influences [1]. Concerns about fetal well-being, future child care, or changes in the family dynamics following childbirth may cause anxiety among expectant mothers [2]. A pathological fear of childbirth that impacts or disrupts a pregnant woman’s daily life is known as fear of childbirth (FOC) or tokophobia [3]. FOC falls on a diagnostic spectrum, with worries about childbirth at one end and phobic levels of childbirth fear at the other [4]. The pooled prevalence of tokophobia globally is 14%, with a range between 3.7 and 43% [5]. The lack of standard criteria for diagnosis, variable assessment tools, and cultural differences contributed to the extensive range of prevalences between countries [4,6].

Numerous factors, such as maternal depression, anxiety, inadequate social support, miscarriages, and sexual abuse, have been associated with tokophobia [6,7]. In multiparous women, fear of childbirth is linked to past traumatic delivery, operative or instrumental birth, emergency obstetric procedures, and birth complications [7,8]. Research has demonstrated a connection between FOC and longer labor, more epidural usage, increased risks of labor dystocia, and emergency caesarean sections [9,10].

Recent data support a link between childbirth fear and adverse birth experiences and post-traumatic stress disorder [11,12]. A bad delivery experience may negatively impact maternal-infant bonding and exclusive breastfeeding [13]. Unfavorable birth experiences raise a woman’s risk of postnatal depression and adversely influence her outlook on future delivery, which can lead to a maternal request for a caesarean section [3,14].

Fear of childbirth is multi-dimensional. The content of childbirth fear varied, according to qualitative studies, and included pain, uncertainty, loss of control, solitude, and risk to both the mother and the child [15]. Standardized instruments to assess tokophobia may be restrictive due to a diverse spectrum of cultural backgrounds, alongside varying perspectives and beliefs around childbirth [4]. The most widely utilized diagnostic tool is the Wijma Delivery Expectancy Questionnaire Part A (WDEQ-A). Despite being valid and reliable across various populations, there were concerns about the cross-cultural applicability of the items [16]. Additionally, exploratory factor analyses of the WDEQ-A in several European cohorts have indicated that the FOC content may vary by nation [9].

Most tokophobia research has been conducted in the developed Scandinavian and European countries [7]. As birth is a multifaceted experience, it is natural for childbirth fear to differ across regions or cultures [17]. Recognizing the causes of tokophobia is essential to developing and implementing effective interventions that will decrease its burden on pregnant mothers. The primary objective of our study was to identify the factors associated with maternal childbirth fear among pregnant women in Asia and secondly, to determine its prevalence in the region. We hope that the results will lead to an improved understanding of tokophobia in Asian women and promote culturally sensitive research on the topic.

## 2. Materials and Methods

This review was carried out in compliance with the Preferred Reporting Items for Systematic Reviews and Meta-Analyses extension for Scoping Reviews (PRISMA-ScR) Checklist [18]. By conducting a scoping review, we aimed to obtain a comprehensive overview of the available evidence on the research topic instead of a systematic review that would produce critically appraised and synthesized results to a specific research question. Nevertheless, a scoping review still requires rigorous and transparent methods similar to those of a systematic review to ensure trustworthy results [19].

We conducted a literature search in September 2023 across three electronic databases (PubMed, Scopus, and Web of Science) using the following search string: (childbirth fear OR “tokophobia” OR “prenatal fear”) AND (risk OR pred* OR “risk factor”). The search window included the period between the databases’ creation and 11 September 2023.

We included all original research papers in English, with the following criteria:

(1) Studies were conducted in Asian countries;
(2) Fear of childbirth was studied as the primary outcome;
(3) Maternal childbirth fear was assessed antenatally;
(4) FOC was measured using a validated scale, not as a subdomain of a larger instrument.

All cross-sectional and cohort studies, meta-analyses, and systematic reviews were included to identify relevant studies.

We excluded articles with the following features:

(1) Letters or commentary, conference abstracts, editorial, and book chapters;
(2) Studies conducted postpartum;
(3) Qualitative or interventional studies.

The literature was organized using EndNote (version 20.6, Clarivate, PA, USA), which helped detect and remove duplicate items. Two authors (I.K.A and A.K.) screened the article titles and abstracts for relevant studies. The full texts of the chosen articles were then obtained for a thorough evaluation in accordance with the inclusion and exclusion criteria. Any discrepancies in study inclusion were resolved following a discussion with a third author (S.S).

A structured Google form was used to gather comprehensive information from the included studies. Authors, year of publication, study design, assessment tools, subject characteristics (gestation, parity, and pregnancy risk), and study outcomes (prevalence, associated factors, and correlations) were retrieved. Microsoft Excel was used to store and analyze the collected data.

Unlike a systematic review, a scoping review does not aim to “synthesize” evidence or compile findings from several studies [20]. As a result, an assessment of methodological limitations or risk of bias of the evidence included within a scoping review is generally not performed [19]. We, however, decided to assess the quality of included studies for the benefit of our readers. Joanna Briggs Institute (JBI) critical appraisal tools for cross-sectional studies were used for this purpose [21]. Each study was scored based on eight criteria, and the quality ranking was allocated as low (less than 33%), medium (33–66%), or high (over 66%) [22].

## 3. Results

The literature search using three electronic databases yielded 2232 items (PubMed = 541, Scopus = 732, and Web of Science = 959). After removing 930 duplicates, we screened the titles and abstracts of 1302 items. We excluded 1218 articles for various reasons and subjected 84 papers to full-text screening. We initially identified 46 eligible studies that discussed various factors. We chose to publish our findings in two different articles due to the large volume of data. Our first published manuscript involved 26 studies that discussed variables from the following categories: (1) demographics, (2) clinical, (3) healthcare service, (4) childbirth education and information, and (5) COVID-19 [23].

We feel that the psychosocial and spiritual elements related to childbirth deserve to be highlighted, as they are occasionally disregarded in favor of clinical aspects. This paper will therefore focus on the important psychosocial and spiritual components (23 articles), which are divided into the following categories: (1) psychiatric symptoms or disorder, (2) psychological determinants, (3) perception and experience, (4) relationships and support, (5) spirituality, and (6) COVID-19. A total of seventeen studies were discussed in both papers.

**Figure 1:**
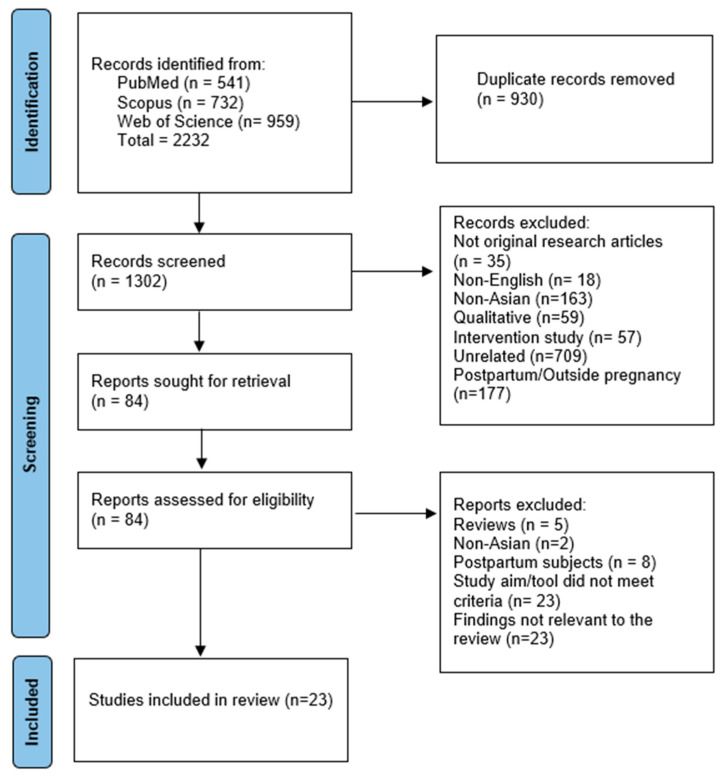
PRISMA flowchart demonstrating the selection of articles.

Figure 1 demonstrates the process of article selection for our review. Twenty-three studies were labeled “findings not relevant to review,” indicating that the results could have been published in our first paper or were unsuitable for current publication.

Table 1 presents the studies included in our review, which were published between 2014 and 2023. Most of the research was carried out in Turkey and China. Four papers from Iran were included in our review, while the other studies originated from Vietnam, Pakistan, Indonesia, and Japan. All of the studies were cross-sectional in design and involved a total of 10,538 women. The majority of research employed the WDEQ-A or Childbirth Attitude Questionnaire (CAQ) to measure fear of childbirth. Three studies focused on nulliparous subjects, two studies focused on high-risk women, and one study involved multiparas. Various instruments were used to identify the correlations between maternal childbirth fear and psychosocial factors. The overall prevalence of childbirth fear in this review ranged from 56.6% to 82.1%, with different cut-off scores used to assess FOC severity. The various threshold score for the FOC diagnosis is presented in a separate appendix (Table A2).

The quality assessment of included studies is presented in Table A1 (Appendix A). We found that most studies were of high quality, and only four (17%) were ranked as having medium quality, with scores ranging between 38% and 100%.

Table 2 presents the independent psychosocial factors associated with maternal tokophobia, as determined by multivariable logistic or linear regression analysis. Factors with significant correlations to tokophobia (Table 3) are also discussed in this review.

Additional information on the included studies, i.e., study population background risk and the studies’ conclusions/recommendations, is presented in the Appendix A (Table A2).

### 3.1. Psychiatric Symptoms/Disorder

A history of psychiatric disorder is an independent predictor of tokophobia, with an increased risk of more than sixfold [27]. Takegata et al. also demonstrated a positive correlation between the lifetime prevalence of mental illness and maternal FOC [40]. Various studies have consistently reported positive associations between maternal depression and tokophobia [29,43,44,45]. Similarly, anxiety is a significant predictor of tokophobia among Asian women [17,29,43,44]. Marcelina et al. found that women who expressed childbirth-related anxiety were three times more likely to experience tokophobia (adjusted odds ratio, AOR 3.37, 95% CI 1.4–7.9, *p* = 0.005) [35]. Zhou et al. reported that pregnancy-related stress was also a significant predictor of tokophobia [45].

### 3.2. Psychological Determinants

Childbirth self-efficacy has a protective effect against tokophobia, as reported by multiple studies from China and Turkey [17,32,33,39]. Maternal psychological well-being was also negatively correlated with fear of childbirth [24,26]. Maternal ability to cope with childbirth with a positive coping style, in particular, protects against childbirth fear [31,43]. Maternal resilience and happiness were also protective against the fear of childbirth [24,33]. Intolerance to uncertainty significantly increased the risk of tokophobia [31].

### 3.3. Perception, Experience & Spirituality

Past pregnancy experience was negatively correlated with childbirth fear [34]. Nguyen et al. demonstrated that maternal concern for pregnancy-related physical changes was positively linked to greater tokophobia [38]. Spiritual well-being significantly reduced the fear of childbirth [28]. Similarly, studies from Iran found a negative correlation between spiritual intelligence and tokophobia [24,37].

### 3.4. Support and Relationship

Social support was significantly protective against childbirth fear [32,45], as was spousal support [31]. Lack of spousal support was linked to greater FOC [33], while dissatisfaction towards the husband’s support increased the risk of tokophobia by almost twelvefold [35]. Zhang et al. demonstrated the role of good family function in reducing childbirth fear (β = −0.32, *p* < 0.049) [44]. In contrast, fear of childbirth was two and a half times more common among women who had experienced intimate partner abuse (AOR 2.47, 95% CI 1.01–6.02) [36].

### 3.5. COVID-19 Pandemic

Research conducted during the COVID-19 pandemic revealed a positive correlation between fear of childbirth and COVID-19 obsession (r = 0.216, *p* < 0.001) and anxiety (r = 0.138, *p* = 0.013) [25]. A higher degree of tokophobia was also linked to maternal fear of contracting COVID-19 [41,42].

## 4. Discussion

To our knowledge, this scoping review is the first to demonstrate a comprehensive overview of the psychosocial determinants of childbirth fear among Asian women. Most studies (83%) were ranked as high quality. Despite the heterogeneity of the study populations in our review—some of the studies were focused on nulliparous women—the results showed similarities to groups with mixed parity, indicating the strength of the variables [26,35,37]. The majority of the studies utilized WDEQ-A and CAQ to diagnose FOC. Although both tools have been validated in various countries, doubts persist about the validity of translated versions due to cultural nuances [4,46]. Differences in cut-off scores and translations may introduce bias or misclassification of FOC levels. According to a recent Chinese study, WDEQ and CAQ assessments of the same subjects result in differing FOC incidences (29.9% vs. 43.9%). Although both instruments demonstrated good reliability and validity, WDEQ performed better due to its comprehensive evaluation capacity [47].

Recent statistics showed an increasing trend of tokophobia since 2015, with a worldwide prevalence of severe childbirth fear of 16% (95% CI 14–16%) [48]. The prevalence of high to severe FOC in our review was higher (range 3.9% to 46.0%). Developing countries’ social, political, and health systems differ from those of wealthy industrialized nations, which may cause these disparities [48]. Additionally, societal norms and institutions, religious beliefs and practices, and ethnicity all have an impact on tokophobia [49].

Our review found that maternal psychiatric illness was linked to a greater risk of tokophobia. A systematic review by Dencker et al. involving studies from the USA, Australia, and Scandinavian countries confirmed the correlation between FOC and mental health issues [7]. Rouhe et al. reported that individuals with tokophobia more often received psychiatric care than those without FOC (54.0% vs. 33.6%, *p* < 0.001) [50]. Söderqvist et al. reported that the likelihood of childbirth fear in women with previous psychological problems was increased by almost twofold (OR 1.7, 95% CI 1.1–2.5) [51].

We found that maternal depression and anxiety were independent predictors of tokophobia, in line with other studies from Western countries [51,52,53,54,55]. According to a Norwegian study, the most significant risk of childbirth fear was seen in women who suffered from both anxiety and depression. (OR 11.0, 95% CI 6.6–18.3) [55]. The transition from pregnancy to delivery and parenthood can be stressful to expectant mothers, and the related uncertainties are a source of maternal anxiety [17]. Previous studies identified several dimensions of prenatal anxiety that include pregnancy, childbirth, hospitalization, parenting the child, and general psychiatric symptomatology [56]. While the prevalence of depressive symptoms is consistent throughout pregnancy and comparable to the postpartum period, pregnancy-related anxiety is more prevalent during the third trimester [56,57]. Pregnancy stress was also an independent predictor of childbirth fear among Asians. Zhou et al. found that tokophobia among Chinese women increased with late gestation, which can be attributed to the change in stress levels throughout pregnancy, with expectant mothers in their last trimester expressing greater concern about their capacity to give birth [45].

Self-efficacy is a dynamic cognitive process that allows individuals to evaluate their capacity to handle different situations and carry out appropriate behaviors [58]. Women with low self-efficacy tend to be less motivated throughout labor and delivery. They believe that childbirth is a difficult endeavor and question their capacity to undergo labor [59]. Our review found consistent evidence of the protective effect of high childbirth self-efficacy against tokophobia in Asian women. Previous research from Sweden and Australia was in line with our findings and confirmed the associations between low self-efficacy and severe FOC [60,61].

Interestingly, Huang et al. demonstrated self-efficacy’s mediating role in the relationship between maternal resilience and FOC [33]. The degree of a woman’s stress resilience affects her emotional suffering, coping strategies, and how she perceives a stressor. High levels of mental and physical well-being are typically associated with high stress resilience [62]. A study from China confirmed the negative relationship between maternal resilience and tokophobia [33]. High-resilience expectant mothers make the most of their psychological resources to increase their mental fortitude in coping with childbirth. This makes them feel more confident about the upcoming delivery, hence reducing their fear [33].

Pregnant women’s self-confidence was also linked to their optimistic stress-reduction strategies [63]. A study from our review demonstrated that women who adopt positive coping styles experienced less tokophobia. Han et al. concluded that positive coping mechanisms help pregnant mothers deal with pregnancy-related stress and are linked to increased mental resilience [31]. The authors also found that intolerance to uncertainty (IU) among pregnant women was positively correlated with fear of childbirth, in line with previous findings [64]. Higher levels of IU are typically associated with increased psychological distress and uncertainty avoidance [31].

Psychological well-being is described as spiritual, emotional, and mental well-being [65] and is linked to positive emotions that influence both physical and mental health [28]. Studies from Turkey and Iran demonstrated a negative correlation between maternal psychological well-being and childbirth fear [26,28]. Individuals with positive psychological well-being can handle challenges despite not being in the best mental and emotional states [66].

Bilgic et al. also found a significant inverse relationship between spiritual well-being and tokophobia [28]. Spirituality is the most significant determinant in problem-solving behaviors. Previous research indicates that people with high degrees of spirituality are better able to handle problems and life situations [67]. Women in a spiritual well-being state are more capable of handling the challenges of childbirth, are less anxious throughout labor, and have greater self-confidence [24,68]. Previous research also supported the positive effect of spiritual intelligence towards general health and happiness [69], in line with the Iranian study in our review. Abdollahpour et al. also demonstrated a negative correlation between maternal happiness and tokophobia. The authors concluded that increasing the level of spiritual intelligence in pregnant women can increase their happiness and reduce their fear of childbirth [24].

Qualitative research has shown that intense childbirth fear in multiparous women may have resulted from previous adverse birth experiences, which left them feeling scared, alone, and doubtful about their capacity to give birth [70]. Norwegian data indicated that the risk of childbirth fear was increased in women with a previous negative birth experience (OR 4.8, 95% CI 2.8–8.3). Additionally, Storksen et al. discovered that women’s tokophobia risk doubled after experiencing an obstetric complication (OR 2.6, 95% CI 1.2–5.5), and the risk was even greater if the women experienced multiple complications [71]. These findings were supported by a large study involving six European countries that reported a fivefold increase in childbirth fear among multigravidas with adverse birth experiences (AOR 5.11, 95% CI 4.07–6.42) [53]. In contrast, the Turkish study in our review found a lower level of childbirth fear among women who remembered their past births negatively [34]. Korucku et al. explained that culturally, motherhood increases a woman’s social value and elevates her status within her social circle [72]. This could inspire mothers to put their unpleasant experiences behind them and welcome their upcoming pregnancy with joy and appreciation [34]. The mother’s prior delivery experience might also boost her confidence and ability to handle challenging situations such as labor and childbirth [72], hence making her less fearful in the subsequent pregnancy.

Our review confirmed the protective effect of spousal and social support against maternal childbirth fear, in keeping with previous studies [52,53,73]. European data reported that women in a stable relationship (married or cohabiting) were less likely to suffer from severe tokophobia (AOR 0.64, 95% CI 0.45–0.87) [53]. Previous research indicated that mothers who received spousal support perceived motherhood favorably and were better equipped to handle stress during pregnancy [74]. Pregnant women’s self-efficacy and fear of childbirth are mediated by partner support [16], which is positively correlated with maternal psychological well-being (r = 0.48, *p* < 0.001 [26].

Intimate partner violence (IPV), on the other hand, is a positive predictor of tokophobia. Moghaddam Hossieni et al. reported that Iranian women who experienced physical IPV were 2.5 times at risk of suffering from childbirth fear (AOR = 2.47; 95% CI 1.01–6.02) [36]. Similarly, pregnant Turkish women who had been exposed to IPV reported a greater level of tokophobia compared to mothers who had never experienced violence [75]. European studies also demonstrated higher levels of tokophobia among women with a history of abuse (regardless of type), either in childhood or as adults [48,53,76].

Having social support throughout pregnancy is crucial in preserving maternal mental health. According to Fisher et al., social connections can strengthen women’s perceptions that labor is a physiological and manageable process, thus improving their psychological well-being and decreasing FOC [77]. Family support, including knowledge and firsthand accounts of childbirth, encourages expectant mothers to stay optimistic about the delivery [78]. Zhou et al. found that perceived social support mediates the relationship between antenatal depression and FOC. Enhancing maternal social support will lower depressive symptoms, psychological issues brought on by stressful life situations, and, ultimately, the fear of childbirth [79].

Several studies in this review were conducted during the COVID-19 pandemic. Pregnant women experienced psychological distress and a poor state of well-being as a result of the disease outbreak and nationwide lockdown [80]. Stressors that contributed to pregnancy-related anxiety during the worldwide pandemic included the possibility of COVID-19 transmission to oneself and the fetus, as well as concerns about delivery, loss of household income, and marital conflict [81]. Pregnant women were also more susceptible to severe COVID-19 complications due to pregnancy-related physiological and immunity changes [82]. Unsurprisingly, maternal tokophobia and fear of contracting the COVID-19 infection are positively correlated [41,42]. A study from Turkey demonstrated that COVID-19 obsession and anxiety among pregnant women resulted in increased levels of childbirth fear. Aksoy et al. also reported that expectant mothers who closely followed news on COVID-19 were twice as likely to suffer from tokophobia [25], which concurred with recent evidence on the positive correlation between COVID-19 media consumption and psychological distress [83].

### 4.1. Clinical Implications

Antenatal screening for FOC and related psychological symptoms, as well as domestic violence, is crucial to identify the high-risk group so that timely intervention can be offered to pregnant women [31,33,36]. Discussions with multiparas about their past delivery experiences and current birth expectations will guide women-centred midwifery care [34]. Assisting expectant mothers to identify their social support system in early pregnancy, alongside facilitating their partners through prenatal training and care, will help to alleviate maternal pregnancy stress and increase their childbirth self-efficacy [17,35,45]. Therapy that focuses on emotional regulation that includes spiritual components may also benefit women with tokophobia.

Additionally, health care providers play a crucial role in raising awareness of tokophobia and offering customised advice and care—such as prenatal education, pregnancy yoga, and mindfulness training—that increase a mother’s confidence during childbirth [33]. Optimizing perinatal mental health through early screening and intervention that focuses on emotional support, psychoeducation, or behavioral strategies is also important for positive mother-infant bonding and healthy child development [84]. High prevalence of perinatal mental health disorders in low and middle-income countries (LMIC) places a heavy burden on maternal and infant well-being. Therefore, developing and integrating perinatal mental health support into existing maternal and child health services should be a national priority, in line with the WHO’s Sustainable Development Goal 3 (health and well-being) [85,86].

### 4.2. Strengths and Limitations

Our study is the first to review research specifically focused on Asian women comprehensively. We discussed the independent psychosocial predictors of maternal childbirth fear derived from multivariable regression analysis. The analytical approach we chose to demonstrate significant independent variables, as part of evidence “mapping,” may be useful for further research/systematic reviews. Our assessment shed further light on Asian mothers’ experiences with tokophobia, which is different from those of the more commonly researched Western populations.

There are some limitations to our review. Firstly, our results may not be truly representative of all Asian countries due to a paucity of data from the Indian subcontinent, the Middle East, and other countries in Southeast Asia. Second, the cross-sectional design of the research in our review precludes the determination of a causal association between the variables. Thirdly, excluding other study designs, such as qualitative studies, from our review limits the depth of the psychosocial analysis and insight into childbirth from various cultural perspectives. Finally, results should be interpreted cautiously because of the population’s social structure, economic development, and ethnic diversity.

## 5. Conclusions

Our review highlighted the psychosocial determinants of tokophobia among Asians. As childbirth becomes more medicalized nowadays, the associated emotional components are often overlooked [28]. Our review highlighted the important psychosocial determinants of FOC among Asians. Screening for psychological symptoms, domestic violence, and negative birth experiences should be part of the FOC assessment. Partner involvement in prenatal care and education should be highly encouraged to strengthen women’s support. A comprehensive psychological intervention for women with FOC should also include a spiritual component. Improving perinatal mental health is crucial for optimal long-term maternal and infant health. Finally, culturally driven qualitative research is necessary to better understand maternal expectations of childbirth and to help develop practical interventions that enhance maternal psychological and spiritual well-being.

## Figures and Tables

**Table 1:** Included studies.

No	Author	Year	Country	Study Aim: To Investigate	Gestation in Weeks; Parity (Special Risk)	Total Number (N)	FOC Assessment Tool	FOC Prevalence (Level)	Other Assessment Tools
1	Abdollahpour [24]	2018	Iran	The relationship between spiritual intelligence and happiness and FOC	20–34; all	245	CAQ	NA	SISRI; OHQ
2	Aksoy [25]	2023	Turkey	The relationship between COVID-19 obsession and anxiety and FOC in high-risk pregnancies	≥20; all (high-risk women)	326	FOBS	NA	CAS; OCS
3	Anjum [26]	2022	Pakistan	The relationship among women’s fear of childbirth, well-being, and partner support	>35; nullip	100	WDEQ-A	NA	Partner Support Questionnaire; Well-being in Pregnant Women Questionnaire
4	Barat [27]	2023	Iran	To screen for FOC and associated factors	≥20; all	600	WDEQ	29.2% Severe	PPRQ; Pregnancy History; Body Image Questionnaire; Pelvic Injury Questionnaire
5	Bilgic [28]	2021	Turkey	The relationship between FOC and psychological and spiritual well-being	Any; all	338	WDEQ-A Turkish	70.1% Overall 19.8% High 13.9% Clinical	PWBS; SWBS
6	Citak [17]	2021	Turkey	The psychosocial predictors of FOC in pregnant women	≥28; all	624	WDEQ-A Turkish	20.8% Severe	CBSIE-Short Form; MPSS; STAI-T; PSEQ-Relationship with husband
7	Eroglu [29]	2022	Turkey	The prevalence of FOC and associated factors, including vaginismus, in pregnant women with high/severe FOC	24–40; all	407	WDEQ-A Turkish	82.1% Overall 32.2% High 13.8% Severe	BDI; BAI; GRISS-vaginismus subscale
8	Gao [30]	2015	China	FOC and its predictors among Chinese women	≥28; all	353	CAQ Chinese	NA	STAI; CBSEI (Chinese)
9	Han [31]	2022	China	The associations between coping styles, intolerance of uncertainty, and FOC	24–40; all	969	CAQ Chinese	67.8% Overall	IUS-12; SCSQ
10	Hou [32]	2022	China	FOC and its predictors in re-pregnant women after caesarean section	≥28; all	358	CAQ Chinese	NA	CBSEI-32 (Chinese); SSRS
11	Huang [33]	2021	China	The prevalence and predictors of FOC among Chinese women	≥11; all	646	CAQ Chinese	NA	CBSEI-32; CD-RISC-10
12	Korukcu [34]	2019	Turkey	The effects of previous birth experience(s) on the FOC in the current pregnancy	28–40; multip	309	WDEQ-A	69.6% Overall 19.1% Severe	Past pregnancy experience: happily/proudly/in pain/fear
13	Marcelina [35]	2019	Indonesia	The predictors of childbirth fear among Indonesian primigravida	≥28; nullip	126	WDEQ-A	45.2% Severe	MAT; PSS; PRAQ-R2
14	Moghaddam Hossieni [36]	2017	Iran	The prevalence of intimate partner violence (IPV) and its prediction of FOC	≥14; all	174	rFDQ	61.5% Overall	CTS2; STAI
15	Mohamadirizi [37]	2017	Iran	The relationship between spiritual intelligence and fear of delivery in low–risk women	≥28; nullip	220	CAQ	NA	Spiritual Intelligence Self-Report Inventory
16	Nguyen [38]	2021	Vietnam	The FOC and willingness to pay for fear-prevention services in pregnant women	ns; all	900	FOBS	NA	MSPSS; Concerns about physical changes; PICSS
17	Qiu [39]	2019	China	The status of FOC and its associated factors among nulliparous women in China	ns; all	1039	CAQ Chinese	NA	Modified General Perceived Self-Efficacy Scale
18	Takegata [40]	2014	Japan	The relationship between FOC and sense of coherence (SOC)	37; all	226	WDEQ-A Japanese	NA	SOC
19	Tiryaki [41]	2022	Turkey	The fear of birth and COVID-19 in high-risk pregnant women	20–40; all (high-risk women)	238	FOBS	NA	FCV-19S
20	Ulu [42]	2022	Turkey	The relationship between fear of childbirth, fear of COVID-19, and marital adjustment	ns; all	382	WDEQ-A	NA	Fear of COVID-19 Scale; RDAS
21	Yildrim [43]	2023	Turkey	The influences of anxiety and depression on FOC	≥ 28; all	501	WDEQ-A	72.7% Overall 46.0% Moderate	BDI; BAI
22	Zhang [44]	2023	China	The prevalence and risk factors of FOC among pregnant women in the third trimester of pregnancy	≥28; all	535	CAQ Chinese	56.6% Overall 3.9% Severe	APGAR; CAHPS; Oslo 3-item social support scale; GSES; GAD-7; PHQ-9, ISI; WHOQOL-8
23	Zhou [45]	2021	China	The prevalence and risk factors for fear of childbirth	14–41; all	922	CAQ	70.3% Overall 6.0% Severe	MSPSS, EPDS, PPS
						10,538			

NA not available; ns not specified; nullip nullipara; multip multipara. Assessment tools abbreviations: APGAR Adaptation Partnership Growth and Resolved; ASI Anxiety Sensitivity Index; BAI Beck Anxiety Inventory; BBS Birth Beliefs Scale; BDI Beck Depression Inventory; BFI Big Five Inventory; BSRI BEM Gender Roles Inventory; CAHPS Consumer Assessment of Healthcare Providers and Systems; CAS COVID-19 Anxiety Scale; CAQ Childbirth Attitude Questionnaire; CBSEI Childbirth Self-Efficacy Inventory; CBSEI-32 32 item-Childbirth Self-Efficacy Inventory; CBSIE-Short Short form of Childbirth Self-Efficacy Inventory; CD-RISC-10 Connor-Davidson Resilience Scale; CTS2 Revised Conflict Tactics Scale; DIS Discomfort Intolerance Scale; DTS Distress Tolerance Scale; EPDS Edinburgh Postnatal Depression Scale; FCV-19S Fear of COVID-19 Scale; FOBS Fear of Birth Scale; GAD-7 Generalized Anxiety Disorder-7; GRISS Golombok Rust inventory of sexual satisfaction; GSES General Self-Efficacy Scale; ISI Insomnia Severity Index; IUS-12 Intolerance of Uncertainty Scale-12; MAT Marital Adjustment Test; MSPSS Multidimensional Scale of Perceived Social Support; NUPDQ The Revised Prenatal Distress Questionnaire; OCS COVID-19 Obsession Scale; OHQ Oxford Happiness Questionnaire; PADQ Pakistan Anxiety and Depression Questionnaire; PHQ-9 Patient Health Questionnaire-9; PICSS Perinatal Infant Care Social Support Scale; PPS Pregnancy Pressure Scale; PRAQ-R2 Pregnancy-Related Anxiety Questionnaire; PPRQ Pregnancy Risk Questionnaire; PSEQ Prenatal Self-Evaluation Questionnaire; PSS Perceived Stress Scale; PWBS Psychological Well Being Scale; RAS Relationship Assessment Scale; RDAS Revised Dyadic Adjustment Scale; rFDQ Revised version of the Fear of Vaginal Delivery Questionnaire; SCSQ Simplified Coping Style Questionnaire; SCS-SF Self-Compassion Scale-Short Form; SISRI Spiritual Intelligence Self Report Inventory; SOC Sense of Coherence Scale; SSRS Social Support Rating Scale; STAI State-trait anxiety inventory; STAI-T State-Trait Anxiety Inventory-Trait; STICSA State The Trait Inventory for Cognitive and Somatic Anxiety; SWBS Spiritual Well Being Scale; WHOQOL-8 EUROHIS-QOL 8-item index; VAS Visual Analog Score.

**Table 2:** Independent psychosocial factors (based on multi-variable regression analysis).

Factors	Independent Factors	Studies
***Psychiatric Symptoms/Disorder***		
Psychiatric disorder	History of psychiatric disorder (AOR 6.86, *p* = 0.020)	Barat 2023 [27]
Depression	Depression score (β = 0.441, *p* < 0.001)	Yildrim 2023 [43]
	Depressive symptoms (β = 0.220, *p* < 0.001)	Zhou 2021 [45]
	Depression (AOR 1.11, *p* < 0.001)	Eroglu 2022 [29]
	Depression (β = 0.30, *p* = 0.027)	Zhang 2023 [44]
Anxiety	State-anxiety (β = 0.24, *p* = 0.002), Trait-anxiety (β = 0.27, *p* = 0.001)	Gao 2015 [30]
	Anxiety (β = 0.239, *p* < 0.001)	Yildrim 2023 [43]
	Trait-anxiety (β = 0.287, *p* < 0.001)	Citak 2021 [17]
	Anxiety (AOR 1.03, *p* = 0.045)	Eroglu 2022 [29]
	Anxiety (β = 0.50, *p* = 0.001)	Zhang 2023 [44]
	Childbirth-related anxiety (AOR 3.37, *p* = 0.005)	Marcelina 2019 [35]
Stress	Pregnancy stress (β = 0.394, *p* < 0.001)	Zhou 2021 [45]
***Psychological Determinants***		
Childbirth Self-efficacy	Childbirth self-efficacy (β = −0.790, *p* < 0.001)	Qiu 2019 [39]
	Childbirth self-efficacy (β = −0.495, *p* < 0.001)	Huang 2021 [33]
	Childbirth self-efficacy (β = −0.463, *p* < 0.001)	Citak 2021 [17]
	Childbirth self-efficacy (β = −1.284, *p* < 0.001)	Hou 2022 [32]
Psychological well-being	Psychological well-being (β = −0.273, *p* < 0.001)	Bilgic 2021 [28]
Maternal coping	Coping with childbirth (β = −0.088, *p* = 0.041)	Yildrim 2023 [43]
	Negative coping style (β = 0.261, *p* < 0.001); Positive coping style (β = −0.135, *p* < 0.001);	Han 2022 [31]
Resilience	Maternal resilience (β = −0.305, *p* < 0.001)	Huang 2021 [33]
Uncertainty	Intolerance to uncertainty (β = 0.277, *p* < 0.001)	Han 2022 [31]
***Perception and Experience***		
Concern about body changes	Concerns about physical changes (β = 0.20, *p* < 0.01)	Nguyen 2021 [38]
***Spirituality***		
Spirituality	Spiritual well-being (β = −0.304, *p* < 0.001)	Bilgic 2021 [28]
***Support and Relationship***		
Social support	Perceived social support (β = −0.091, *p* = 0.019)	Zhou 2021 [45]
	Social support (β = −0.353, *p* = 0.001)	Hou 2022 [32]
Spousal support	Lack of spousal support (β = 0.93, *p* < 0.001)	Huang 2021 [33]
	Relationship with partner (β = −0.059, *p* = 0.032)	Zhou 2021 [45]
	Satisfaction on husband’s support; dissatisfaction (AOR 11.96, *p* = 0.001)	Marcelina 2019 [35]
	Full spousal support (β = −0.305, *p* < 0.001	Han 2022 [31]
Family Function	Good family function (β = −0.32, *p* < 0.049)	Zhang 2023 [44]
Partner violence	Physical intimate partner violence (AOR = 2.47, *p* < 0.05)	M Hossieni 2017 [36]

AOR adjusted odds ratio.

**Table 3:** Significant correlations between FOC and psychosocial and spiritual factors.

Factors	Correlations with FOC	Studies
***Psychiatric Disorder***		
Psychiatric disorder	Lifetime prevalence of mental illness (r = 0.16, *p* = 0.01)	Takegata 2014 [40]
***Psychological Determinants***		
Psychological well-being	Psychological well-being (r = −0.49, *p* < 0.001)	Anjum 2023 [26]
Happiness	Happiness (r = −0.69, *p* < 0.05)	Abdollahpour 2018 [24]
***Perception and Experience***		
Previous birth experience	Past pregnancy experience (r = −0.17, *p* < 0.05)	Korukcu 2019 [34]
***Spirituality***		
Spirituality	Spiritual intelligence (r = −0.73, *p* < 0.05)	Abdollahpour 2018 [24]
	Spiritual intelligence (r = −0.163, *p* = 0.025)	Mohamadirizi 2017 [37]
***COVID-19***		
COVID-19 infection	COVID-19 Anxiety (r = 0.138, *p* = 0.013)	Aksoy 2023 [25]
	COVID-19 Obsession (r = 0.216, *p* < 0.001)	Aksoy 2023 [25]
	COVID-19 Fear (r = 0.268, *p* < 0.001)	Tiryaki 2022 [41]
	COVID-19 Fear (r = 0.130; *p* = 0.011)	Ulu 2022 [42]

## Data Availability

Data sharing does not apply to this article, as no datasets were generated or analyzed during the current study.

## Abbreviations

The following abbreviations are used in this manuscript:

FOC	fear of childbirth
WDEQ-A	Wijma Delivery Expectancy Questionnaire Part A
CAQ	Childbirth Attitude Questionnaire
IU	intolerance to uncertainty
IPV	intimate partner violence

## Appendix A

**Table A1:** Assessment of included cross-sectional studies using Joanna Brigg’s Institute (JBI) critical appraisal tools.

JBI Critical Appraisal Checklists for Cross-Sectional Studies	Abdollahpour, 2018 [24]	Aksoy, 2023 [25]	Anjum, 2022 [26]	Barat, 2023 [27]	Bilgic, 2021 [28]	Citak, 2021 [17]	Eroglu, 2022 [29]	Gao, 2015 [30]	Han, 2022 [31]	Hou, 2022 [32]	Huang, 2021 [33]	Korucku, 2019 [34]	Marcelina, 2019 [35]	Moghaddam Hossieni, 2017 [36]	Mohamadirizi, 2017 [37]	Nguyen, 2021 [38]	Qiu, 2019 [39]	Takegata, 2014 [40]	Tiryaki, 2022 [41]	Ulu, 2022 [42]	Yildrim, 2023 [43]	Zhang, 2023 [44]	Zhou, 2021 [45]
Were the criteria for inclusion in the sample clearly defined?	Yes	Yes	Yes	Yes	Yes	Yes	Yes	Yes	Yes	Yes	Yes	Yes	Yes	Yes	Yes	Yes	Yes	Yes	Yes	NC	Yes	Yes	Yes
Were the study subjects and the setting described in detail?	Yes	Yes	Yes	Yes	Yes	Yes	NC	Yes	Yes	Yes	Yes	Yes	NC	NC	NC	Yes	Yes	Yes	Yes	NC	Yes	Yes	Yes
Was the exposure measured in a valid and reliable way?	Yes	Yes	NC	Yes	Yes	Yes	Yes	Yes	Yes	Yes	Yes	Yes	Yes	Yes	Yes	Yes	Yes	Yes	Yes	Yes	Yes	Yes	Yes
Were objective, standard criteria used for measurement of the condition?	Yes	Yes	NC	Yes	Yes	Yes	Yes	Yes	Yes	Yes	Yes	Yes	Yes	Yes	Yes	Yes	Yes	Yes	Yes	NC	Yes	Yes	Yes
Were the confounding factors identified?	No	Yes	No	Yes	No	Yes	Yes	Yes	Yes	Yes	Yes	NC	NC	Yes	NC	Yes	Yes	Yes	No	No	Yes	Yes	Yes
Were strategies to deal with confounding factors stated?	No	Yes	No	Yes	No	Yes	Yes	Yes	Yes	Yes	Yes	NC	NC	Yes	NC	Yes	Yes	Yes	No	No	Yes	Yes	Yes
Were the outcomes measured in a valid and reliable way?	Yes	Yes	NC	Yes	Yes	Yes	Yes	Yes	Yes	Yes	Yes	Yes	NC	Yes	NC	Yes	Yes	Yes	Yes	Yes	Yes	Yes	Yes
Was appropriate statistical analysis used?	Yes	Yes	Yes	Yes	Yes	Yes	Yes	Yes	Yes	Yes	Yes	Yes	Yes	Yes	Yes	Yes	Yes	Yes	Yes	Yes	Yes	Yes	Yes
Total Quality Assessment Score for each study (based on six criteria)	75% High	100% High	38% Med	100% High	75% High	100% High	88% High	100% High	100% High	100% High	100% High	75% High	50% Med	88% High	50% Med	100% High	100% High	100% High	75% High	38% Med	100% High	100% High	100% High

NC: unclear; NA: not applicable; Med: medium; quality ranking allocation: low (less than 33%), medium (33–66%), or high (over 66%).

**Table A2:** Included Studies’ Conclusions and Recommendations.

No	Author, Year	Study Population Background Risk	FOC Tool	FOC Threshold Score	Conclusion/Recommendations
1	Abdollahpour, 2018 [24]	Low-risk, no psychiatric diagnosis	CAQ	FOC > 32	Increasing the level of spiritual intelligence in pregnant women can lead to an increase in their happiness and reduce FOC. FOC can be prevented via maternal training about the components of spiritual intelligence.
2	Aksoy, 2023 [25]	High-risk pregnancy	FOBS	FOC > 50	Women with high-risk pregnancies may experience COVID-19 anxiety, which may worsen FOC. Psychosocial interventions focusing on COVID-19 anxiety are warranted for these women.
3	Anjum, 2022 [26]	Low-risk	WDEQ-A	NS	Partner support is essential to overcome FOC and help increase the well-being of first-time pregnant women.
4	Barat, 2023 [27]	Women with CS indications excluded	WDEQ	FOC > 85	It is necessary to design a comprehensive antenatal training program to reduce FOC among women with risk factors, i.e., having an academic education, a self-employed spouse, a history of infertility, and psychiatric illness.
5	Bilgic, 2021 [28]	Low-risk, no psychiatric diagnosis	WDEQ-A Turkish	High: 66–84 Clinical > 85	There was a negative correlation between psychological (PWB) and spiritual well-being (SWB) and FOC. SWB was a partial mediating variable in PWB and FOC relationship. PWB and SWB of pregnant women should be evaluated in order to reduce FOC.
6	Citak, 2021 [17]	Low-risk, no psychiatric diagnosis	WDEQ-A Turkish	Severe > 85	The psychosocial variables predicting FOC were self-efficacy and trait anxiety. Spousal support is a mediator between self-efficacy and FOC. Individualized education programs and delivery preparation training, based on maternal psychosocial needs, may lead to better coping with FOC.
7	Eroglu, 2022 [29]	No previous CS	WDEQ-A Turkish	High: 66–84 Severe > 85	Depression and anxiety level, educational level, access to information on delivery during pregnancy, presence of medical disease, and expression of FOC were predictors of high/severe FOC. Pregnant women with high/severe FOC also had a significantly higher vaginismus score. Assessment of FOC and associated risk factors, including vaginismus, during pregnancy, will enable the identification of risk groups and the creation of support programs.
8	Gao, 2015 [30]	Low-risk, no previous CS	CAQ Chinese	NS	State-anxiety, trait-anxiety, age, and previous miscarriage were predictors of childbirth fear among pregnant Chinese women. The health-care professionals should be sensitive toward childbirth fear and assess pregnant women’s age, educational level, and previous miscarriage to identify the pregnant women who may have severe childbirth fear.
9	Han, 2022 [31]	Low-risk, no psychiatric diagnosis	CAQ Chinese	FOC > 28 Moderate: 40–51 Severe: 52–64	Primiparas, unplanned pregnancy, few spousal supports, intolerance of uncertainty, and negative coping styles were significant risk factors in FOC. Regular screening assessment of perinatal psychological symptoms, such as a high level of intolerance of uncertainty and negative coping styles, should be adopted to reduce the risk of FOC.
10	Hou, 2022 [32]	Low-risk, no psychiatric diagnosis	CAQ Chinese	NS	The number of CSs, experience with previous CS, childbirth self-efficacy, and social support were predictors of FOC among re-pregnant women after CS in China. Healthcare professionals need to identify these women with high-risk of FOC and provide appropriate services during pregnancy.
11	Huang, 2021 [33]	Low-risk, no previous CS, no psychiatric diagnosis	CAQ Chinese	NS	Age, gestational age, parity, spousal support, resilience, and childbirth self-efficacy were predictors of FOC. Healthcare professionals should pay close attention to FOC and implement targeted interventions in accordance with these predictors, especially resilience and childbirth self-efficacy
12	Korukcu, 2019 [34]	Low-risk, no psychiatric or chronic disease	WDEQ-A	Moderate 61–84 Severe ≥ 85	Counterintuitively, there is a negative relationship between the previous birth experience and childbirth fear. Childbirth fear can persist through the postpartum period, and health care professionals need to address FOC through sensitive discussions with women about the birthing experience and their thoughts concerning future births.
13	Marcelina, 2019 [35]	NS	WDEQ-A	NS	Satisfaction with the husband’s support and maternal anxiety were important predictors of childbirth fear. It is recommended for health care providers to facilitate husbands throughout antenatal care to support their wives in preventing FOC.
14	Moghaddam Hossieni, 2017 [36]	Low-risk, no psychiatric diagnosis	rFDQ	Severe fear ≥ 6	All pregnant women experiencing physical violence had a higher chance of FOC. Screening programs for FOC and intimate partner violence (IPV) need to be implemented particularly in nulliparous women. Providing continuity of midwifery care and family therapy may be strategies for early support to reduce IPV to pregnant women.
15	Mohamadirizi, 2017 [37]	Low-risk, no medical/psychiatric diagnosis	CAQ	NS	High spiritual intelligence in pregnant women can reduce the fear of childbirth.
16	Nguyen, 2021 [38]	Low-risk, no chronic condition or fetal loss	FOBS	NS	FOC was associated with age of partner; previous pregnancy complications; attitudes toward different aspects of childbirth delivery; satisfactions with friends, parents, and siblings’ care; and information support. Individualized psychological counseling and information-seeking guidance should be provided appropriately for multiparous and nulliparous women to reduce fear and improve the acceptability of the prevention services.
17	Qiu, 2019 [39]	NS	CAQ Chinese	NS	Education levels, self-rated health status, self-efficacy levels, and use of pregnancy-related smartphone applications were predictors of FOC among pregnant women in China. Healthcare professionals should focus on the above factors in identifying pregnant women with FOC and implementing targeted interventions.
18	Takegata, 2014 [40]	Low-risk, not planning CS	WDEQ-A Japanese	Severe: 85–99 Phobia > 100	Sense of coherence (SOC) was negatively linked with antenatal FOC. High SOC works as a resiliency factor that helps pregnant women cope with the stress of their upcoming childbirth and reduces FOC.
19	Tiryaki, 2022 [41]	High-risk (maternal and fetal), no psychiatric diagnosis	FOBS	FOC ≥ 50	Prenatal anxiety was prevalent among high-risk pregnant women who required routine anxiety screening and psychosocial support during the COVID-19 pandemic.
20	Ulu, 2022 [42]	Unspecified	WDEQ-A	NS	Fear of childbirth was positively correlated to fear of COVID-19 and negatively correlated to marital adjustment. Psychological support to pregnant women is beneficial to reduce their fears and to improve marital adjustment by including the spouses in the process.
21	Yildrim, 2023 [43]	Low-risk, no previous CS	WDEQ-A	NS	Lack of prenatal education, inability to cope with childbirth, and depression and anxiety were significant predictors of FOC. Health professionals can provide individualized care, education, and counseling services by taking into account the effects of depression and anxiety so that expectant women’s fear of childbirth can be reduced.
22	Zhang, 2023 [44]	No psychiatric disorder	CAQ Chinese	FOC ≥ 28 Moderate: 40–51 Severe: 52–64	Significant FOC risk factors were prolonged exposure to electronic screens, nullipara, previous CS, preference to sour food or dislike greasy food, worrying about delivery without family members, family function, doctor-patient communication, anxiety, and depression. There is an urgent need to develop interventions to reduce FOC in the third trimester of pregnancy, with particular attention to those with risk factors.
23	Zhou, 2021 [45]	Low-risk, no psychiatric diagnosis	CAQ	FOC ≥ 28 Moderate: 40–51 Severe: 52–64	FOC showed a positive correlation with pregnancy-related stress and depressive symptoms and a negative correlation with social support. Screening for FOC and helping pregnant women identify a support system early in pregnancy could reduce a woman’s stress level and the severity of depression.

CAQ: Childbirth Attitude Questionnaire; CS: caesarean section; FOBS: Fear of Birth Scale; NS: not specified; rFDQ: Revised version of the Fear of Vaginal Delivery Questionnaire; WDEQ-A: Wijma Delivery and Expectation Scale-A.
